# Factors Associated With Trial Completion and Adherence in App-Based N-of-1 Trials: Protocol for a Randomized Trial Evaluating Study Duration, Notification Level, and Meaningful Engagement in the Brain Boost Study

**DOI:** 10.2196/16362

**Published:** 2020-01-08

**Authors:** Jason R Bobe, Jacqueline Buros, Eddye Golden, Matthew Johnson, Michael Jones, Bethany Percha, Ryan Viglizzo, Noah Zimmerman

**Affiliations:** 1 Institute for Next Generation Healthcare Department of Genetic and Genomic Sciences Icahn School of Medicine at Mount Sinai New York, NY United States

**Keywords:** crossover trials, mobile apps, adherence, nootropics, N-of-1 trials, attrition, study duration, usability, motivation, cognition, mHealth, randomized controlled trial

## Abstract

**Background:**

N-of-1 trials promise to help individuals make more informed decisions about treatment selection through structured experiments that compare treatment effectiveness by alternating treatments and measuring their impacts in a single individual. We created a digital platform that automates the design, administration, and analysis of N-of-1 trials. Our first N-of-1 trial, the app-based Brain Boost Study, invited individuals to compare the impacts of two commonly consumed substances (caffeine and L-theanine) on their cognitive performance.

**Objective:**

The purpose of this study is to evaluate critical factors that may impact the completion of N-of-1 trials to inform the design of future app-based N-of-1 trials. We will measure study completion rates for participants that begin the Brain Boost Study and assess their associations with study duration (5, 15, or 27 days) and notification level (light or moderate).

**Methods:**

Participants will be randomized into three study durations and two notification levels. To sufficiently power the study, a minimum of 640 individuals must begin the study, and 97 individuals must complete the study. We will use a multiple logistic regression model to discern whether the study length and notification level are associated with the rate of study completion. For each group, we will also compare participant adherence and the proportion of trials that yield statistically meaningful results.

**Results:**

We completed the beta testing of the N1 app on a convenience sample of users. The Brain Boost Study on the N1 app opened enrollment to the public in October 2019. More than 30 participants enrolled in the first month.

**Conclusions:**

To our knowledge, this will be the first study to rigorously evaluate critical factors associated with study completion in the context of app-based N-of-1 trials.

**Trial Registration:**

ClinicalTrials.gov NCT04056650; https://clinicaltrials.gov/ct2/show/NCT04056650

**International Registered Report Identifier (IRRID):**

PRR1-10.2196/16362

## Introduction

### Background

The purpose of this study is to evaluate factors that may impact study completion and adherence in the context of app-based N-of-1 trials. A common challenge for digital research studies is the poor engagement of users [1]. The “law of attrition,” as Eysenbach described it in 2005, is of special concern to app-based N-of-1 studies. Unlike a conventional, two-arm, randomized controlled trial (RCT) where a minimum sample size is calculated to detect a treatment effect that also takes into account an expected rate of attrition across the enrolled population, N-of-1 trials operate at the level of the individual, and there is 1 participant per trial. Typically, in an N-of-1 trial, an individual alternates between treatments (ie, “multiple crossover”), and outcomes are measured during each treatment period [2,3]. If there is attrition and the participant fails to complete an N-of-1 trial, there is no result for that individual.

Moreover, if a participant completes an N-of-1 trial, the result may still fail to achieve a level of statistical meaningfulness, especially for shorter trial durations. In this way, any person that aims to design an N-of-1 trial that is capable of informing decision-making for treatment selection for a single individual must strike the right balance between the ease of trial completion and the generation of meaningful results. Therefore, we aim to collect evidence about critical factors that may impact rates of study completion in the context of app-based N-of-1 trials. We hope these findings will inform the design of future app-based N-of-1 trials and improve the adoption of N-of-1 methods and tools.

### N-of-1 Trials

N-of-1 trials create an opportunity for individuals to optimize treatment selection more systematically. In contrast, “therapy by trial” is a more common practice in both wellness and clinical medicine and is where individuals begin a therapy and monitor outcomes, often without much formal structure. If a treatment is deemed ineffective or introduces intolerable treatment burdens, a change to the treatment is made. N-of-1 trials are an alternative approach designed to help individuals make more objective, data-driven treatment choices.

Usually, in an N-of-1 trial, an individual alternates between treatments, and outcomes are measured during each period [2,3]. Where feasible, treatments may be blinded or placebo-controlled. Outcomes are measured at baseline and each treatment period. At the end of the trial, outcome measurements for each treatment are compared, and a treatment is selected. N-of-1 trials are particularly relevant in contexts where evidence for treatment efficacy is weak or where treatment response is known to vary across patient populations [2]. N-of-1 trials may also be deployed to answer other common treatment questions, such as optimal dosage or whether a symptom is associated with a treatment’s side effects [2]. In what is considered a landmark paper for modern N-of-1 trials, Guyatt and colleagues applied this methodology to compare two treatments in a single patient with uncontrolled asthma and discovered that one treatment made the patient feel worse [4,5].

N-of-1 trials are not useful in every treatment context. Treatments with rapid onset and minimal washout are ideal candidates for N-of-1 trials, whereas curative treatments or treatments with cumulative effectiveness (eg, antidepressants) are not. N-of-1 trials are suitable for individuals with chronic or stable conditions. For example, one might compare melatonin versus herbal tea for chronic insomnia, and another might compare the effectiveness of two topical creams for persistent acne [2].

### Hypotheses

The primary hypothesis is that shorter trials will have higher rates of completion compared to longer trials. The null hypothesis is that no correlation exists between study completion and study duration. We also hypothesize that higher rates of study completion will be achieved with more frequent reminders to complete study tasks, in the form of app-based notifications, especially since the Brain Boost Study requires participants to complete tasks during a specific window of time each day during the trial. The null hypothesis is that no correlation exists between study completion and notification level.

## Methods

### Study Setting

**Figure 1:**
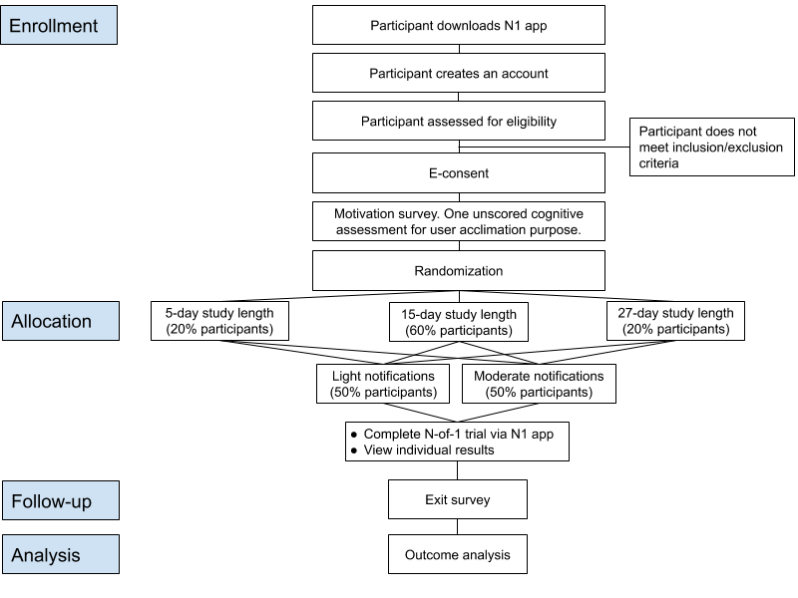
Participant flow through the Brain Boost Study using the N1 app. E-consent: electronic consent.

All study activities are conducted through the N1 app, a smartphone iOS app distributed via the Apple App Store. We will primarily recruit participants via social media and through messages to online communities where there is documented interest in the topics of nootropics, supplements, medical science, or health technology. The informed consent process takes place remotely through the app, as described elsewhere [6]. Participants may contact the study staff at any time via email with questions or concerns. We have a designated health care professional on the study team to follow up on participant-reported, health-related concerns that are related to their participation in the Brain Boost Study. Participant flow through the study is described in Figure 1.

### Eligibility Criteria

Adults aged 18 or older who have an iPhone running iOS 11.0 or later, consume caffeine, and live in the United States are eligible to enroll in the study. Exclusion criteria include anyone with reason to believe that consuming caffeine may be harmful to their health, are pregnant, or are breastfeeding. If a participant is unsure about whether they have a health issue that prevents them from consuming caffeine, they are advised to consult their doctor but are still eligible for the study.

### Study Design

This study aims to evaluate a novel platform for conducting single-patient, multiple crossover studies (N-of-1 trials). For purposes of clarity, this protocol describes our methods to assess factors associated with study completion, along with several exploratory measures and analyses. The study design, interventions, cognitive assessment instruments, and methods for evaluating whether there is a detectable treatment effect for an individual participant enrolled in the Brain Boost Study based on their performance on the cognitive assessments are described elsewhere [6]. Participants enrolled in the Brain Boost Study follow a treatment schedule, guided by a mobile app (N1 app), where they alternate between the two treatments: caffeine (treatment A) or caffeine combined with L-theanine (treatment B) during a prespecified window of time. Participants are also asked to complete an app-based cognitive assessment during a prespecified window of time each day. The daily cognitive assessment includes three separate cognitive tests: the Stroop Test, the Remote Associates Test, and the Trailmaking Test [6]. They complete their tasks during one baseline period (where no treatment is assigned), and during four treatment periods where either treatment A or treatment B is assigned according to counterbalanced block design. Participants are not compensated, and the app is free to use. Only after a trial is completed does a participant see their results from the cognitive assessments.

### Randomization

The study duration is either 5, 15, or 27 days. Participants will be randomized into study duration according to the allocation of 20%, 60%, and 20%, respectively. The uneven allocation to study duration was chosen out of consideration for the trade-offs between various study lengths. While we hypothesize that 5-day study lengths are more likely to be completed, they are also less likely to generate a meaningful result due to the small number of outcome measures. On the other hand, 27-day studies are more likely to generate a meaningful result with more repeated outcome measures, but we hypothesize that they are more likely to result in early withdrawal. Allocating more people to the 15-day study is a reasonable compromise between these two extremes, which is reflected by our decision to allocate more participants into this group while maintaining a sufficient sample size for each group to assess our primary outcome measure, as described below.

Notification level is defined as the frequency participants receive reminders to complete study tasks and is either light or moderate. Participants are randomized into the notification level, such that 50% of participants are in each group. Participants randomized into the light notification group receive two notifications per day: one reminder to take their treatment and one reminder to complete the assessment (see Multimedia Appendix 1). Participants randomized into the moderate notification level receive 4-5 notifications per day: 2 reminders to take their treatments and 2-3 reminders to complete their assessment (see Table 1).

**Table 1:** Notification levels for the Brain Boost Study.

Notification level	Treatment reminders	Assessment reminders
Light	At treatment time	At assessment time
Moderate	15 minutes before At treatment time	15 minutes before At assessment time 15 minutes before the end of assessment window, only if the assessment is incomplete at this time

### Measures

#### The Proportion of Studies Completed

The primary outcome of interest is the proportion of studies completed. A study is considered complete if a participant reaches the end of a trial without a study failure (involuntary withdrawal) or voluntary withdrawal. A study failure occurs when there is insufficient data generated during baseline or any treatment period due to missed treatments (self-reported) or incomplete assessments. Participants must complete all assigned treatment and assessment tasks for 1/3 of the days in each period to avoid study failure and involuntary withdrawal, except for the 5-day study where all tasks must be completed each day because there is only one day per period. For the 15-day and 27-day trials, we inevitably expect participants to miss some tasks during these periods, so we wanted to allow for some nonadherence. While the requirement of task completion for 1/3 of the days in each treatment period is somewhat arbitrary, the choice of 1/3 of the days means that, at a minimum, completed 15-day trials will at least have the same amount of data as completed 5-day trials and are also likely to have more. We applied the same criteria to the 27-day trial so that the criteria for study failure are uniform across all three study durations. It is also worth noting that the 3 study lengths are primarily dictated by our randomized, counterbalanced, crossover design that defines N-of-1 trials: NABBA or NBAAB, where N is baseline, A is treatment period A, B is treatment period B, and AB or BA is a block. The minimum N-of-1 trial for a counterbalanced design with two blocks is five days if you have only one day per treatment period and one day of baseline.

We anticipate that a 27-day trial approaches what is likely to be the maximum number of days we could expect people to participate with reasonable adherence for this study, which requires daily tasks. The 15-day trial splits the difference between these two extremes. Put another way, we chose to vary treatment period lengths by one day for the 5-day trial, three days for the 15-day trial, and five days for the 27-day trial. We could have selected a different design, such as treatment periods that are 1, 2 and 3 days long rather than 1, 3 and 5 days long for each group; however, to evaluate the impact of study durations and notification levels on completion rates, we reasoned it would be better to have a more extensive spread across the three groups.

#### Adherence

Treatment adherence is recorded as true for days when a participant completes a cognitive assessment and does not report treatment nonadherence. A participant is instructed to report any treatment nonadherence (eg, a missed treatment or the use of caffeine on a baseline day) in the app during the trial. At the end of the trial, participants are prompted to review a summary of their treatment adherence and may, if necessary, record missed treatments at this time as well. Assessment adherence is recorded as true when a participant completes all three cognitive tests in a daily cognitive assessment according to the user-specified schedule. Otherwise, assessment nonadherence is recorded (for both incomplete assessments and assessments that were not started). Daily adherence is recorded as true when a participant achieves both treatment adherence and assessment adherence for a given day during the trial.

We will also measure trial adherence, which is defined as the proportion of total actions completed by a participant during a trial. Here total actions are defined as the number of requested treatment actions (to take a treatment or abstain from all treatments) plus the number of requested assessment actions (take a cognitive assessment) during a trial. During the baseline period, participants take two actions per day in the form of abstaining from treatments and completing one cognitive assessment. During a treatment period, participants take two actions per day in the form of taking one treatment plus completing one cognitive assessment. The total number of actions requested, and the minimum number of actions required for each study duration are outlined in Table 2.

**Table 2:** Total number of actions requested, and the minimum number of actions required for each study duration.

Study duration	Total number of actions requested	Minimum number of actions required to avoid study failure
5-day study	10	10
15-day study	30	10
27-day study	54	22

#### Motivation and Notification Levels

Before the trial begins, we will ask participants to self-report their motivation level to learn their results on a 5-point ordinal scale (see Multimedia Appendix 2). Also, participants will be randomized into two notification levels (light or moderate). However, since Apple does not allow iOS apps submitted to the App Store to require that users allow app notifications, we will also record the number of participants that turn off notifications. We anticipate that the number of individuals that turn off notifications will be rare, in part due to a warning message we included as a modal in the app that discourages this behavior, due to the likelihood of failure to complete study tasks according to the user-specified daily schedule (see Multimedia Appendix 1).

#### Proportion of Trials That Yield Statistically Meaningful Results

For each study duration, we will also measure the proportion of completed N-of-1 trials that yield statistically meaningful results as determined by the N1 app, for the comparisons: (1) caffeine versus baseline; and (2) caffeine plus L-theanine versus baseline. As described elsewhere, the N1 app considers a trial to have yielded a statistically meaningful result if the coefficient of treatment effect is significantly different from zero at the 80% confidence level in at least one of the three cognitive tests (eg, if taking caffeine relative to baseline, with or without L-theanine, produces an effect on cognitive performance measured by at least one of the three cognitive tests) [6]. The 80% confidence level is arbitrary, and we anticipate future versions of the N1 app will allow individuals to select the confidence level they seek.

#### Exit Survey

We will also invite enrolled participants to provide feedback about the N1 app and the Brain Boost Study through an optional exit survey administered at the time of study completion, during voluntary withdrawal, or at study failure via an automated email with a link. The anonymous survey will not be linked with individual user accounts. The exit survey includes a modified version of the 2-item Usability Metric for User Experience (UMUX-Lite) and up to 15 optional questions related to the participant experience with the app and the study (see Multimedia Appendix 3) [7]. The survey will be conducted in a browser outside of the N1 app.

#### Bug Reports and App Crashes

We will record the number of software bug reports submitted anonymously by users through a third-party tool linked from inside the N1 app. We will also record the number of app crashes recorded from those users who have opted-in to share their diagnostics and app usage information with app developers in App Store Connect, an administrative platform for managing and monitoring iOS apps submitted to the Apple App Store [8].

#### Other Collected Data

We collect sex (male, female, other) and year of birth (YYYY). For each participant in a trial, we will also timestamp planned and completed actions, and record the following dates: planned trial start and end dates, dates of adherence and nonadherence (treatment and assessment adherence), and dates of early withdrawal or completion.

### Power and Sample Size

The study will be sufficiently powered (>80%; alpha=0.05) if 640 individuals begin the study, are randomized into 3 study lengths (5, 15, or 27 days) according to a randomization percent allocation of 20%, 60%, 20%, and achieve rates of completion of 30%, 20%, and 10%, respectively (see Table 3).

**Table 3:** The minimum number of participants we aim to enroll in each study duration.

Study duration	Randomization allocation (%)	Participants who begin study (n)	Estimate of completion rate (%)	Participants who complete study (n)
5-day study	20	128	30	38
15-day study	60	384	20	77
27-day study	20	128	10	13
Total	100	640	60	128

For the sample size estimate, the Hsieh sample size correction for multiple logistic regression was used, which assumes correlation among covariates and therefore adjusts the sample size accordingly [9]. We estimated this correlation to be equal to 0.25. With these assumptions, 97 completed studies are required to discern whether the study length and notification level are associated with the rates of study completion. To estimate how precise we may be, we used sample size tables for each predictor (see Multimedia Appendix 4).

### Data Analysis Plan

#### Primary Analysis

All study data, unless otherwise noted, will be collected through the N1 app, and stored in Health Insurance Portability and Accountability Act (HIPAA)-compliant cloud storage. Statistical analyses will be conducted primarily using R version 3.6 (The R Foundation, Vienna, Austria). We will use a multiple logistic regression model to discern the relationship between study duration and notification level with the proportion of studies completed among participants who begin a study. Participants who begin a study are defined as those who achieve both treatment adherence and assessment adherence for at least one day during the baseline period. Participants that do not begin the study will be removed from the analysis. We will adjust these results for any variance in completion rate by age and sex.

#### Exploratory Analyses

We will also use descriptive statistics to summarize measures collected in the Brain Boost Study. We will compare the proportion of studies completed and trial adherence across the six groups of participants (three study durations and two notification levels), removing from this analysis anyone that turned off notifications. We will also compare the proportion of completed trials that are deemed by the N1 app to demonstrate a meaningful difference in treatment response on any of the cognitive tests, across the three study durations.

We also want to learn about the factors that influence the measure of trial adherence to improve future study designs. We will use Bayesian methods for exploratory analysis to get a baseline for future analyses, which we expect will also use Bayesian methods of analysis. The exploratory analysis will utilize a Bayesian survival-style model with semicompeting risks throughout the study. It will be assumed that a participant is in the study up until the time of study completion, voluntary withdrawal, or involuntary withdrawal (ie, study failure). Daily adherence will be assessed among all participants in the study on a given study day. There will be two types of events considered: (1) daily nonadherence, as a nonterminal repeating event; and (2) early withdrawal (including both voluntary withdrawal and involuntary withdrawal due to study failure) as a terminal (nonrepeating) event. The daily rate of nonadherence and early withdrawal will be modeled as a Poisson process with the rates impacted by several covariates, including study day (number of days since the start of the study, estimated with a random walk prior), notification level (light versus moderate); self-reported motivation level (5-point ordinal scale), day of the week (if distinguishable from study day), and subject-specific shared frailty term.

The cumulative probability of early withdrawal can be used to estimate the inverse probability of study completion. Also, the daily rates of adherence can be used to improve study design for future participants, depending on self-reported motivation and subject-specific characteristics. As in the primary analysis of the study completion rate, we will additionally adjust this analysis of adherence for age and sex.

## Results

### Platform Development and Testing

We have completed extensive internal testing of the N1 app, as well as beta testing on a convenience sample of 12 users. We did not collect completion rates or adherence for the beta testers because the app was still under development at the time that testing was performed. We iteratively improved the N1 app over the past two years through more than 75 builds until we achieved a stable release and a feature set suitable for launch.

### Ethics Approval

The Institutional Review Board at the Icahn School of Medicine at Mount Sinai has approved this study (IRB-18-00343, IRB-18-00789).

### Enrollment

The Brain Boost Study on the N1 app opened enrollment to the public in October 2019.

## Discussion

### Primary Findings

The N1 App aims to facilitate the design, administration, and analysis of N-of-1 trials. The Brain Boost Study will be the first experiment available on the platform that is open to the public. As a wellness-related study that evaluates the effect of commonly used supplements on cognitive performance, this study also provides an opportunity to socialize N-of-1 methods to much broader audiences. While the first intentionally designed crossover treatment trial dates to the late 1700s and N-of-1 trials like how we conceive of them today have been practiced for decades, adoption remains low [10-12]. Opportunities for the public to apply these methods to their treatment dilemmas are similarly scarce outside of a limited number of clinical settings with expertise in the design, administration, and analysis of N-of-1 trials.

Digital tools, like the N1 app, provide a promising new avenue for making N-of-1 trials more accessible. However, many challenges remain, especially around participant engagement and adherence in app-based research [13]. Sustained engagement of participants has been elusive for many digital health studies to date. In a pooled analysis of 8 app-based digital health studies representing over 100,000 participants, 850,000 study days, and 3.5 million app-based tasks, the median time participants engaged in the study during the first 12 weeks was only 5.5 days, and the median time participants performed active tasks was only two days [14]. To deploy an effective app-based N-of-1 trial, one must reconcile many trade-offs that span study design, technology, and user characteristics. To our knowledge, this will be the first study to rigorously evaluate multiple factors associated with study completion and adherence in the context of app-based N-of-1 trials. One other app-based N-of-1 trial platform has recently evaluated usability and user acceptance of an app in the context of pain-related N-of-1 trials [15,16]. Our findings should be of significant interest to practitioners seeking to design N-of-1 trials using similar methods and tools to support data-driven treatment choices.

### Strengths and Limitations

N-of-1 trials are designed as for-benefit trials that promote informed decision-making among individuals that participate in them [3]. As such, this study of factors that influence study completion in the context of N-of-1 trials is unlikely to be generalizable to other types of digital health studies (eg, observational studies, RCTs) where there is no likelihood of personal benefit. However, insights from this study may apply to future trials in the N1 app and other app-based N-of-1 trial platforms.

The N1 app was designed to be flexibly adapted to other N-of-1 trials that, by definition, will share a few common elements, including notifications to keep a participant on track with a schedule of alternating treatments and regular outcome measures. We anticipate that even within app-based N-of-1 trials, there may be marked differences in adherence and completion rates depending on the nature of the trial and the characteristics of the target population, such as the magnitude of decisional conflict an individual confronts related to treatment selection or the nature and severity of the underlying health condition, respectively. Moreover, the number and difficulty level of actions requested in an N-of-1 trial is likely to influence completion and adherence rates. The collection of outcome measures through the integration of apps, devices, or wearables that reduce the number of actions required of an individual to complete in app-based N-of-1 trial is an area where digital health research may defy the law of attrition and is a promising future direction.

This protocol requires participants to record treatment nonadherence, rather than to record treatment adherence positively. This choice was made to reduce the number of daily actions required of participants under the assumption that a participant is likely to complete a daily cognitive assessment only in the circumstance where a treatment was taken according to schedule. Since the results of the cognitive assessment are hidden until the completion of the trial, a participant does not stand to benefit by taking a cognitive assessment for reasons that are outside the purpose of the trial.

We anticipate that most participants will schedule their treatments at the beginning of the day since caffeine consumption is a typical morning ritual. This represents two challenges for participant engagement that may not be generalizable across other N-of-1 trials. First, a recent survey of eight app-based digital health studies observed the highest levels of engagement in the evening [14]. Second, the act of abstaining from caffeine consumption for the duration of the baseline period may be perceived as a significant challenge for some individuals in a manner that may be altogether absent were we to be running a trial that compares two treatments of a different nature.

The estimation of completion rates in the total enrolled population for each study duration is a challenge because it not only depends on factors being evaluated in this study but also on recruitment and user characteristics that are uncontrolled outside of very permissive inclusion/exclusion criteria that focus on the safety of caffeine consumption. The novelty of app-based research, for example, may attract people that are more curious about the app or methods than they are committed to learning their results, which is why we included the question about motivation level as an exploratory measure. For example, one recent app-based research study on asthma experienced very high initial recruitment followed by rapid attrition and a small number of highly engaged participants [17]. The relationship between intrinsic motivation and treatment adherence has been examined elsewhere, such as long-term antiretrovirals for HIV/AIDS, weight loss, and various public health initiatives that involve behavior change [18].

One limitation of the collection of only minimal demographic information is that we will not be able to determine if the enrolled study population is representative of the general population outside of age and sex, which will limit the generalizability of our results. The most extensive study to date on retention in digital health studies showed a relationship between age and retention, with older populations having higher retention than younger participants [14]. They observed no relationship between sex and retention [14].

## Appendix

Multimedia Appendix 1 Examples of notifications and permissions in the N1 app.

Multimedia Appendix 2 Motivation survey.

Multimedia Appendix 3 Exit survey.

Multimedia Appendix 4 Sample size tables.

## Abbreviations

HIPAA: Health Insurance Portability and Accountability Act
RCT: randomized controlled trial
UMUX-LITE: 2-item Usability Metric for User Experience

## References

[1] Eysenbach G (2005). The law of attrition. J Med Internet Res.

[2] Nikles J, Mitchell G (2015). The Essential Guide To N-of-1 Trials In Health.

[3] The DEcIDE Methods Center N-of-1 Guidance Panel (Duan N, Eslick I, Gabler NB, Kaplan HC, Kravitz RL, Larson EB, Pace WD, Schmid CH, Sim I, Vohra S), Kravitz RL, Duan N (2014). Design and Implementation of N-of-1 Trials: A User's Guide.

[4] Guyatt G, Sackett D, Taylor DW, Chong J, Roberts R, Pugsley S (1986). Determining optimal therapy--randomized trials in individual patients. N Engl J Med.

[5] Sackett DL (2011). Clinician-trialist rounds: 4. why not do an N-of-1 RCT?. Clin Trials.

[6] Golden E, Johnson M, Jones M, Viglizzo R, Bobe J, Zimmerman N (2019). Measuring the effects of caffeine and L-theanine on cognitive performance: a protocol for self-directed, mobile n-of-1 studies. OSF Preprints.

[7] Lewis JR, Utesch BS, Maher DE (2015). Investigating the Correspondence Between UMUX-LITE and SUS Scores. Design, User Experience, and Usability: Design Discourse.

[8] Apple Inc App Store Connect - Apple Developer.

[9] Hsieh FY, Bloch DA, Larsen MD (1998). A simple method of sample size calculation for linear and logistic regression. Statist. Med.

[10] Kravitz RL, Duan N, Niedzinski EJ, Hay MC, Subramanian SK, Weisner TS (2008). What ever happened to N-of-1 trials? Insiders' perspectives and a look to the future. Milbank Q.

[11] Kravitz RL, Paterniti DA, Hay MC, Subramanian S, Dean DE, Weisner T, Vohra S, Duan N (2009). Marketing therapeutic precision: Potential facilitators and barriers to adoption of n-of-1 trials. Contemp Clin Trials.

[12] Mirza R, Punja S, Vohra S, Guyatt G (2017). The history and development of N-of-1 trials. J R Soc Med.

[13] Druce KL, Dixon WG, McBeth J (2019). Maximizing Engagement in Mobile Health Studies: Lessons Learned and Future Directions. Rheum Dis Clin North Am.

[14] Pratap A, Neto EC, Snyder P, Stepnowsky C, Elhadad N, Grant D, Mohebbi MH, Mooney S, Suver C, Wilbanks J, Mangravite L, Heagerty P, Arean P, Omberg L (2019). Cornell University.

[15] Barr C, Marois M, Sim I, Schmid CH, Wilsey B, Ward D, Duan N, Hays RD, Selsky J, Servadio J, Schwartz M, Dsouza C, Dhammi N, Holt Z, Baquero V, MacDonald S, Jerant A, Sprinkle R, Kravitz RL (2015). The PREEMPT study - evaluating smartphone-assisted n-of-1 trials in patients with chronic pain: study protocol for a randomized controlled trial. Trials.

[16] Odineal DD, Marois MT, Ward D, Schmid CH, Cabrera R, Sim I, Wang Y, Wilsey B, Duan N, Henry SG, Kravitz RL (2019). Effect of Mobile Device-Assisted N-of-1 Trial Participation on Analgesic Prescribing for Chronic Pain: Randomized Controlled Trial. J Gen Intern Med.

[17] Chan YY, Wang P, Rogers L, Tignor N, Zweig M, Hershman SG, Genes N, Scott ER, Krock E, Badgeley M, Edgar R, Violante S, Wright R, Powell CA, Dudley JT, Schadt EE (2017). The Asthma Mobile Health Study, a large-scale clinical observational study using ResearchKit. Nat Biotechnol.

[18] Glenn Cohen I, Fernandez Lynch H, Robertson CT (2016). Nudging Health: Health Law and Behavioral Economics.

