# Supplementary figures and images for: Factors Associated With Trial Completion and Adherence in App-Based N-of-1 Trials: Protocol for a Randomized Trial Evaluating Study Duration, Notification Level, and Meaningful Engagement in the Brain Boost Study
Source: JMIR Res Protoc. 2020 Jan 8;9(1):e16362. doi: 10.2196/16362 (PMC6996754; doi:10.2196/16362)

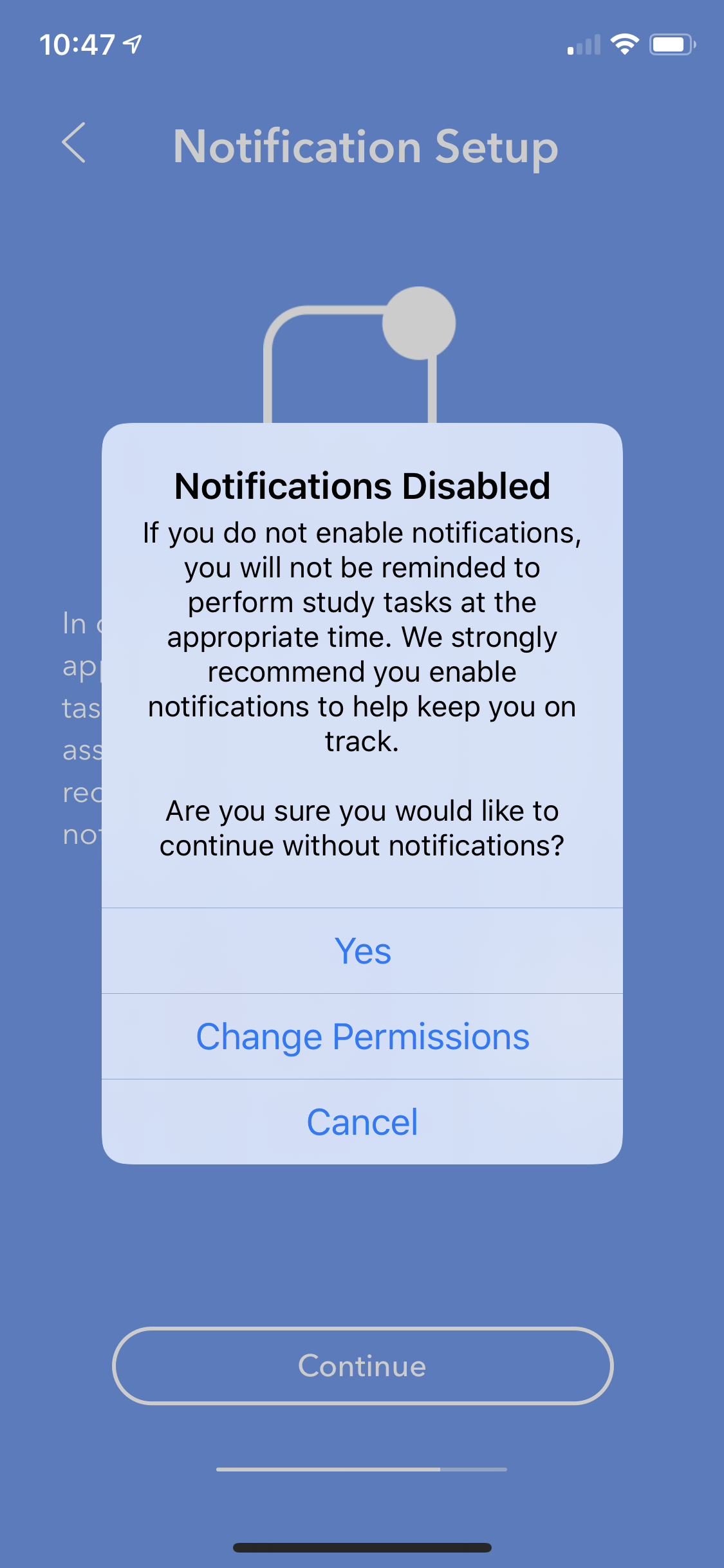

Supplement: Multimedia Appendix 1 [file resprot_v9i1e16362_app1.zip › Notifications/6-notification-warning.PNG]

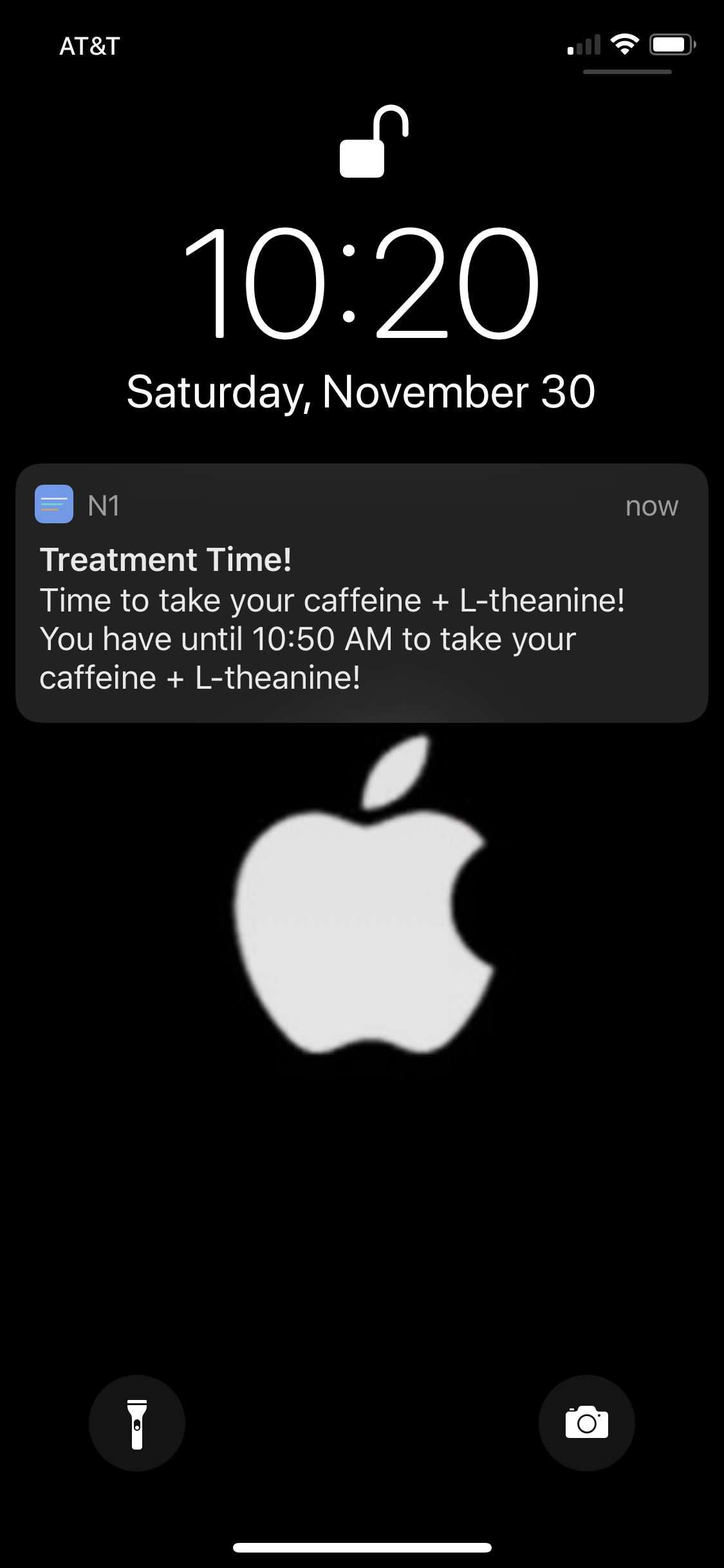

Supplement: Multimedia Appendix 1 [file resprot_v9i1e16362_app1.zip › Notifications/2-treatment.PNG]

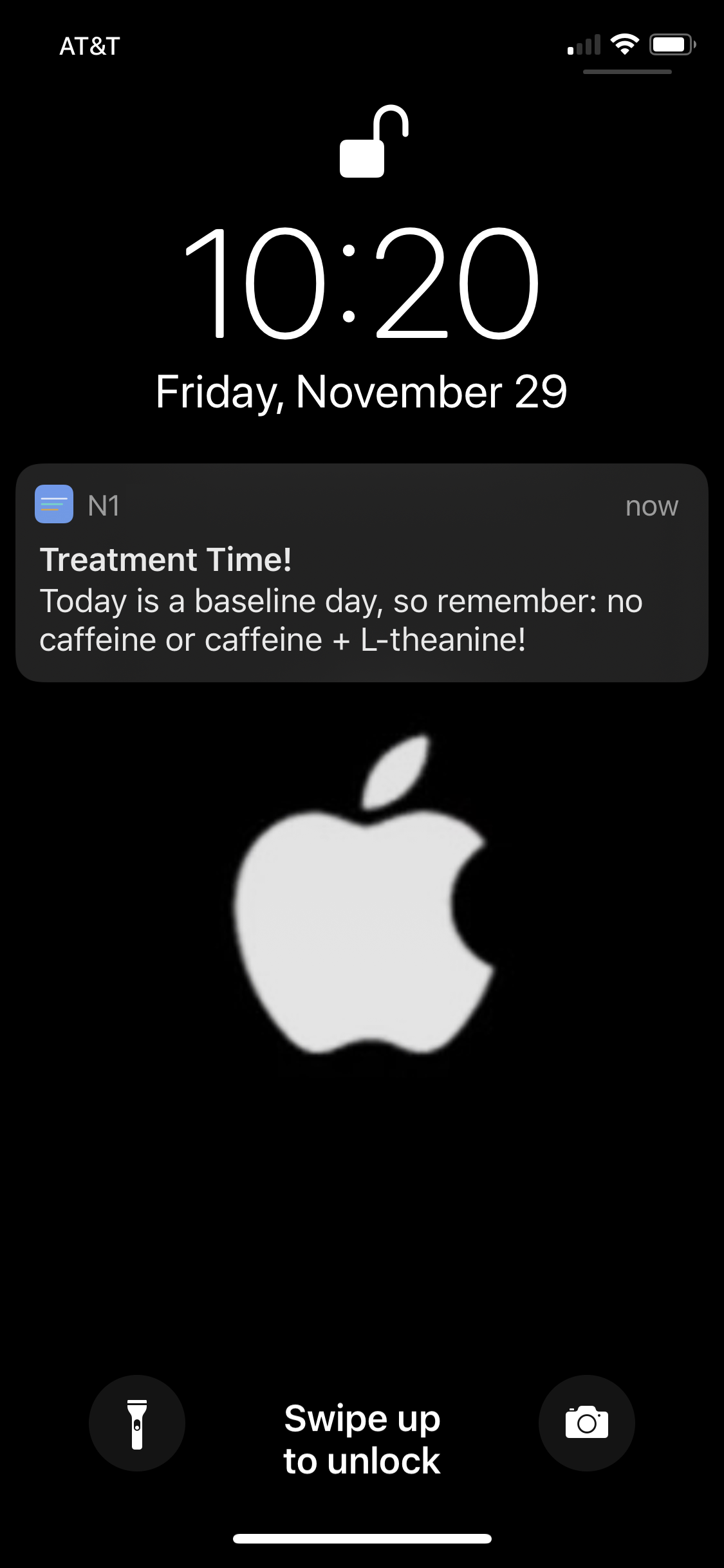

Supplement: Multimedia Appendix 1 [file resprot_v9i1e16362_app1.zip › Notifications/1-baseline.PNG]

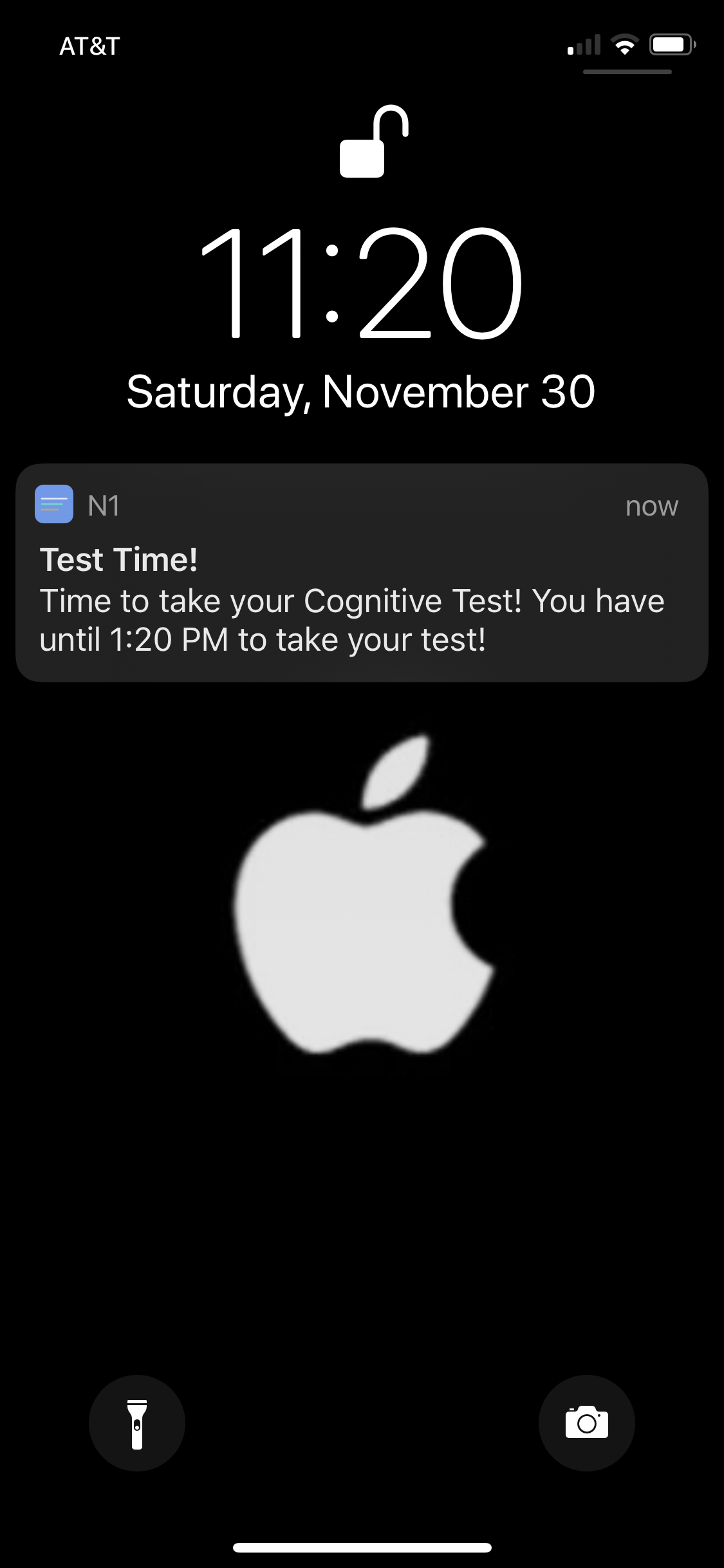

Supplement: Multimedia Appendix 1 [file resprot_v9i1e16362_app1.zip › Notifications/3-cog-test.PNG]

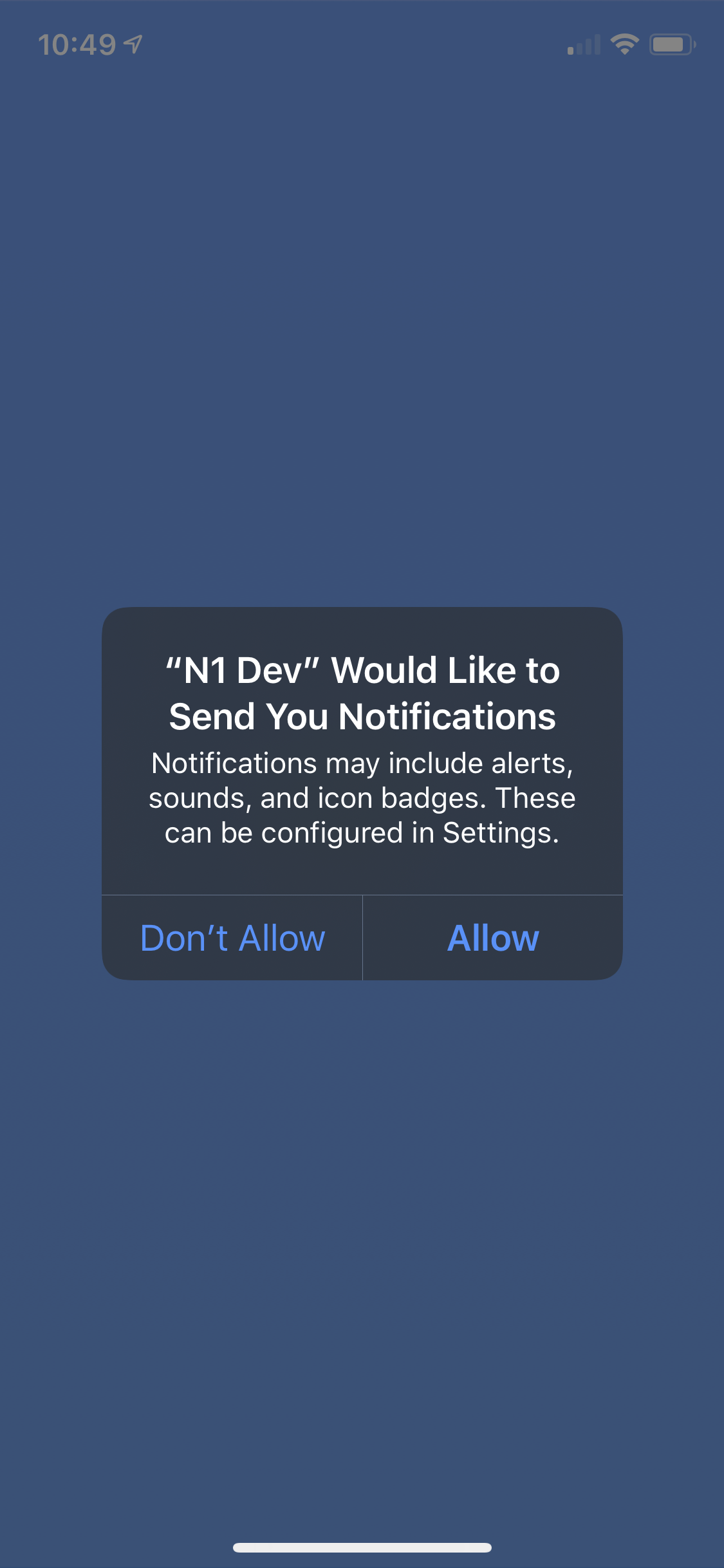

Supplement: Multimedia Appendix 1 [file resprot_v9i1e16362_app1.zip › Notifications/5-allow-notifications.PNG]

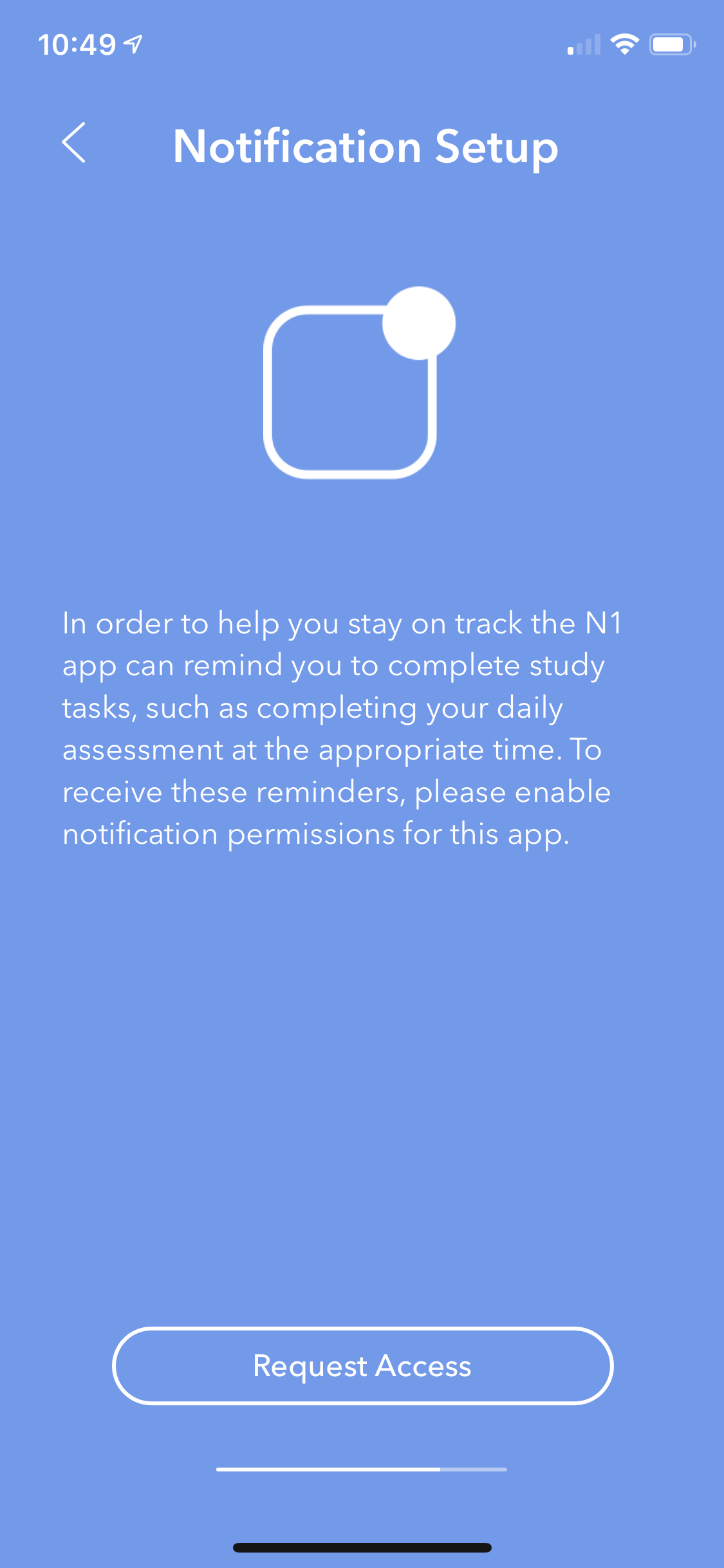

Supplement: Multimedia Appendix 1 [file resprot_v9i1e16362_app1.zip › Notifications/4-notification-setup.PNG]
