# Supplementary material for: Factors Associated With Trial Completion and Adherence in App-Based N-of-1 Trials: Protocol for a Randomized Trial Evaluating Study Duration, Notification Level, and Meaningful Engagement in the Brain Boost Study
Source: JMIR Res Protoc. 2020 Jan 8;9(1):e16362. doi: 10.2196/16362 (PMC6996754; doi:10.2196/16362)
Supplement: Multimedia Appendix 2 [file resprot_v9i1e16362_app2.docx]

# Multimedia appendices

## B. Motivation questionnaire

| I am motivated to learn which treatment improves my cognitive performance   1. Strongly agree 2. Agree 3. Neither agree nor disagree 4. Disagree 5. Strongly disagree |
| --- |
