# Supplementary material for: Factors Associated With Trial Completion and Adherence in App-Based N-of-1 Trials: Protocol for a Randomized Trial Evaluating Study Duration, Notification Level, and Meaningful Engagement in the Brain Boost Study
Source: JMIR Res Protoc. 2020 Jan 8;9(1):e16362. doi: 10.2196/16362 (PMC6996754; doi:10.2196/16362)
Supplement: Multimedia Appendix 3 [file resprot_v9i1e16362_app3.docx]

# Multimedia appendices

## C. Exit survey

| **Welcome to the N1 app user survey**  3 to 5 minute survey asking your experience with the Brain Boost Study using the N1 app. Please do not share any personal information (e.g. name, email, health info) in the survey below. Most questions are optional – you can skip questions that you prefer not to answer.  **1. The N1 app is easy to use.**    1 2 3 4 5 6 7    Strongly Neutral Strongly  Disagree Agree    **2. The capabilities of the N1 app meet my requirements, *i.e.* the app allows me to accomplish the tasks requested for the Brain Boost Study.**    1 2 3 4 5 6 7    Strongly Neutral Strongly  Disagree Agree    3. How did you hear about the project?  [text box]    4. What drew you to the project?  [text box]    5. Did you complete the study from start to finish?  A. Yes  B. No    [IF NO:]  What was the primary reason(s) you didn’t complete the study?  Choose as many as you like  A. Project was not explained properly  B. Getting my treatments was too difficult  C. Study was too long  D. Consent was unclear  E. Staying on top of my treatment was difficult  F. Completing the cognitive tests took too much effort  G. Interface was unclear and difficult for me interpret  H. It was boring  I. The study topic was not of interest to me  J. Other [text box]    Please elaborate on why you didn’t complete the study  [text box]    [IF YES:]    Did the on-boarding process clearly describe what the Brain Boost Study entailed and what you were responsible for doing during the study?  A. Yes it was clear to me  B. Sort of, but I had some questions  C. No, it was confusing  D. Other [text box]    When you returned to the main screen, was it clear where you were in the study and what you had to do? [embed screenshot]  A. Yes  B. No  C. Other [text]    Was it useful to review and have control over your treatment responses in the deep treatment view? [embed screenshot]  A. Yes  B. No  C. Other [text]    Did the app notifications help to keep you on track during the study?  A. Yes  B. No  C. Other [text]    Did you feel you achieved your wellness goal with assistance from the app?  A. Yes  B. No  C. Other [text]    Did the final data visualization help you better understand your wellness goal?  A. Yes  B. No  C. Other [text]    After completing the study, did you share your results via social media or talk to friends about your experience?  A. Yes  B. No  C. Other [text]    Did you learn anything new about your wellness during this study?  A. Yes  B. No  C. Other [text]    What did you find most interesting about the study and app overall?  [Text]    6. Do you have anything else to add that can help us improve the N1 app?  [text] |
| --- |
