# Supplementary material for: Factors Associated With Trial Completion and Adherence in App-Based N-of-1 Trials: Protocol for a Randomized Trial Evaluating Study Duration, Notification Level, and Meaningful Engagement in the Brain Boost Study
Source: JMIR Res Protoc. 2020 Jan 8;9(1):e16362. doi: 10.2196/16362 (PMC6996754; doi:10.2196/16362)
Supplement: Multimedia Appendix 4 [file resprot_v9i1e16362_app4.docx]

# Multimedia appendices

D. Sample size tables

**Sample sizes for Study length: 15-day vs. x**

| **Obs** | **p1** | **p2** | **alpha** | **power** | **totaln_Hsiehadj** |
| --- | --- | --- | --- | --- | --- |
| **1** | 0.25 | 0.33 | 0.05 | 0.8 | 1074 |
| **2** | 0.25 | 0.35 | 0.05 | 0.8 | 700 |
| **3** | 0.25 | 0.40 | 0.05 | 0.8 | 324 |
| **4** | 0.25 | 0.45 | 0.05 | 0.8 | 188 |

**Sample sizes for Study length: 27-day vs x**

| **Obs** | **p1** | **p2** | **alpha** | **power** | **totaln_Hsiehadj** |
| --- | --- | --- | --- | --- | --- |
| **5** | 0.1 | 0.18 | 0.05 | 0.8 | 627 |
| **6** | 0.1 | 0.20 | 0.05 | 0.8 | 424 |
| **7** | 0.1 | 0.25 | 0.05 | 0.8 | 213 |
| **8** | 0.1 | 0.30 | 0.05 | 0.8 | 132 |
| **9** | 0.1 | 0.35 | 0.05 | 0.8 | 91 |
| **10** | 0.1 | 0.40 | 0.05 | 0.8 | 68 |
| **11** | 0.1 | 0.45 | 0.05 | 0.8 | 52 |

**Sample sizes for notification levels (low vs moderate)**

| **Obs** | **p1** | **p2** | **alpha** | **power** | **totaln_Hsiehadj** |
| --- | --- | --- | --- | --- | --- |
| **12** | 0.2 | 0.28 | 0.05 | 0.8 | 951 |
| **13** | 0.2 | 0.30 | 0.05 | 0.8 | 625 |
| **14** | 0.2 | 0.35 | 0.05 | 0.8 | 294 |
| **15** | 0.2 | 0.40 | 0.05 | 0.8 | 174 |

In the tables, p1 and p2 are defined as probability 1 and 2, respectively, of response, i.e. study completion. Also, note that the sample size values in the “totaln_Hsiehadj” column in the tables above refers to the number of individuals that begin a study. For example, if we pick a sample size of 625 individuals, we would be adequately powered to detect a 10% difference (20% vs. 30%) in completion rate between the low vs. moderate notification levels.
